# Whole-Body Vibration Impacts on the Degree of Toe Angle and Its Correlation to the Knee Osteoarthritis Index during Level Walking among Female University Students: A Randomized Controlled Trial

**DOI:** 10.3390/jcm12175735

**Published:** 2023-09-03

**Authors:** Amany E. Abd-Eltawab, Asmaa M. Elbandrawy, Heba B. Ghanem, Hasnaa A. Ebrahim, Mohamed El-Sherbiny, Ateya Megahed Ibrahim, Mohamed Ahmed Eladl, Dalia Mahmoud Abdelmonem Elsherbini

**Affiliations:** 1 Physical Therapy and Health Rehabilitation Department, Faculty of Applied Medical Sciences, Jouf University, Sakaka P.O. Box 2014, Saudi Arabia; aeabdullah@ju.edu.sa; 2 Biomechanics Department, Faculty of Physical Therapy, Cairo University, Giza P.O. Box 12612, Egypt; 3 Department of Physical Therapy for Women’s Health, Faculty of Physical Therapy, Cairo University, Giza P.O. Box 12612, Egypt; asma_elbandrawy@yahoo.com; 4 Department of Physical Therapy for Women’s Health, Faculty of Physical Therapy, Al-Salam University, Tanta P.O. Box 31527, Egypt; 5 Department of Clinical Laboratory Sciences, College of Applied Medical Sciences, Jouf University, Sakaka P.O. Box 2014, Saudi Arabia; hbghanem@ju.edu.sa; 6 Medical Biochemistry Department, Faculty of Medicine, Tanta University, Tanta P.O. Box 31511, Egypt; 7 Department of Basic Medical Sciences, College of Medicine, Princess Nourah bint Abdulrahman University, Riyadh P.O. Box 84428, Saudi Arabia; haebrahim@pnu.edu.sa; 8 Department of Basic Medical Sciences, College of Medicine, AlMaarefa University, Riyadh P.O. Box 71666, Saudi Arabia; msharbini@mcst.edu.sa; 9 Department of Anatomy, Faculty of Medicine, Mansoura University, Mansoura P.O. Box 35516, Egypt; 10 Department of Nursing, College of Applied Medical Sciences, Prince Sattam bin Abdulaziz University, Al-Kharj P.O. Box 11942, Saudi Arabia; a.eleglany@psau.edu.sa; 11 Department of Family and Community Health Nursing, Faculty of Nursing, Port Said University, Port Said P.O. Box 42526, Egypt; 12 Department of Basic Medical Sciences, College of Medicine, University of Sharjah, Sharjah P.O. Box 27272, United Arab Emirates; meladl@sharjah.ac.ae

**Keywords:** level walking, osteoarthritis, toe angle, whole-body vibration

## Abstract

Whole-body vibration (WBV) training is used for ankle rehabilitation as it stimulates muscle spindles to excite tonic vibration reflexes, and improves muscle strength, power, joint proprioception, balance, and flexibility. Thus, this study aims to determine the impact of whole-body vibration on the degree of the toe angle and the correlation between the toe angle and knee osteoarthritis index during level walking among female university students. A randomized controlled trial was conducted with 42 participants divided into two groups. The control group performed only home-based exercise (HBE) after education, and the study group received WBV with HBE. The functional status of participants to predict osteoarthritis was evaluated using the Western Ontario and McMaster osteoarthritis index (WOMAC), where the toe angle degree and WOMAC index were assessed before and six weeks after training. The results showed a significant improvement in the toe-in angle of HBE + WBV compared to the HBE group (*p* = 0.02), and in HBE + WBV, an improvement of the toe angle showed a 43% decrease in the WOMAC index (*p* = 0.001). In conclusion, WBV for the ankle and foot training program positively affected the degree of the toe angle, that directly affected the subtalar and ankle joint mechanics. Trial registration in the Pan African Clinical Trial Registry PACTR202304816093190 (registered retrospectively, date of registration: 18 April 2023).

## 1. Background

The subtalar joint is essential for walking, running, and posture. The mechanisms behind the subtalar joint force are complicated. Chung et al. [1] reported that the subtalar joint is involved a series of complex pronation and supination movements during walking. Pronation at the subtalar joint is a combination of eversion, abduction, and dorsiflexion, which unlocks the foot and causes the medial arch to collapse toward the ground, producing some degree of toe out. At the same time, the lower leg is driven into internal rotation, which causes internal rotation of the hip, which occurs in the first 20% of the gait cycle. Meanwhile, supination at the subtalar joint is a combination of an inversion leading to elevation of the inside border of the foot, causing the toe-in degree to produce external rotation of the lower leg and hip, which occurs after the foot is flat and before the toe-off when inward rotation recurs.

According to the gender variation in the degree of inversion and eversion of the ankle joint, it was reported by Abd-Eltawab et al. [2] that males exhibited greater eversion compared with females, which is insignificant during level walking occurring in the frontal plane. Al-kharaz and Chong [3] proved that females have a more significant ankle range of motion than males in the transverse plane during the stance phase, indicating that the female ankle and foot liability to move toward inversion is more significant than a male’s during walking, which might shift the female leg to become “toe-in”. The position exposes females to sports ankle injuries. Therefore, it was reported by Ristolainen et al. [4] that females, especially female athletes compared to male athletes, are more susceptible to injury. Moreover, it was reported by Quatman et al. [5] that these gender differences might be due to variations in hormone levels. In addition, Fukano et al. [6] reported that females tend to have more ligamentous laxity than males, as females are thought to have better flexibility than males in the ankle joint, causing them to have larger ankle ROM than males.

Whole-body vibration (WBV) is effective neuromuscular training that uses various frequencies of mechanical vibration to induce repetitive muscle contractions to enhance muscle function and proprioception. This type of physical therapy has been widely used to improve muscle strength and performance [7]. WBV training is one of the methods used for ankle rehabilitation [8] as it has an oscillating vibration platform, which stimulates muscle spindles to excite the tonic vibration reflex [9], thus directly enhancing motor control in the ankle [10].

The acute effect of balance on cases with chronic ankle instability was studied by Rendos et al. [11]. They reported that WBV did not considerably improve balance in these cases. However, Chang et al. [12] reported that WBV significantly decreased ankle inversion by 15° during athletic training in females with chronic ankle instabilities. Alongside that, pathological cases with ankle spasticity had a positive value in the degree of muscle tone using WBV that improved their walking abilities [13]. Earlier studies have also postulated an increased frequency of ankle injuries in sport activity players [14,15].

Knee osteoarthritis (OA) is a leading cause of disability and medical expenditures [16]. WBV can improve quadricep muscle strength and physical performance [17], and may activate the alpha-motor neuron, followed by the vibration tonic reflex, by activating muscle spindles, which influences the central mechanism, accounting for the benefits of WBV on knee OA [18]. Gonçalves de Oliveira et al. [19] reported that WBV, compared with control groups, significantly affects muscle strength of the knee extensors and flexors, lower limb extensors, and ankle plantar flexors. Athletes and younger individuals are also susceptible to osteoarthritis (OA), as mentioned by Amoako and Pujalte [20]. Many factors depend on the population and the etiology; injuries, occupational activities, and obesity appear to be the most common causes of OA in young and athletic populations. Moreover, diagnosing athletes and young individuals is sometimes challenging because of their increased pain tolerance.

Research findings indicate the presence of a gap regarding the efficacy of WBV on the toe angle predicting the degree of subtalar joint inversion and eversion and consequently affecting the biomechanics of the ankle joint, especially in younger female students. Therefore, a critical role in designing a rehabilitation program for females, especially in the younger age group, is to prevent further sport-related injuries at the ankle–foot complex. Thus, the current study aimed to investigate the effects of six-week WBV on the dominant leg with toe-in on female feet university students, and assess the correlation between the toe angle and knee osteoarthritis index among them during level walking.

## 2. Methods

### 2.1. Design and Setting

This study was a randomized controlled trial. The CONSORT (Consolidated Standards of Reporting Trials) declaration for randomized controlled trials was followed in the present work [21]. The study was carried out at the Faculty of Physical Therapy, Cairo University, Cairo, Egypt, from January to February 2023.

### 2.2. Procedures

#### Sample Size Calculation

G*Power software (version 3.1.9.2; Heinrich-Heine-Universität, Düsseldorf, Germany) was applied to determine the sample size. The information available between HBE and HBE + WBV was used in detecting the effect size (d) [22,23]. According to the Western Ontario and McMaster osteoarthritis index (WOMAC), the mean ± SD for the two groups was estimated to be 16.96 ± 6.35 and 15.41 ± 5.24, respectively. The effect size (d) (0.27) was applied after reaching an eighty percent power to determine the effect sizes at an alpha level of 5%. The sample sizes for the *t*-test with the two irrelevant groups were 20 for each group. Additionally, past related studies typically reported a sample size of 20 people in each group [24]. Assuming the dropouts, we assigned 50 individuals to participate in the current work.

### 2.3. Participants

Forty-two female university students recruited for the current study had a toe-in degree in at least one of the two feet. All subjects met the following inclusion criteria: 19–21 years old, with a BMI of 20–24 kg/m^2^ and a height of 155–170 cm. All participants had a toe-in degree based on data obtained from The GAIT Rite system. The exclusion criteria were previous trauma, a history of surgery in both legs, congenital anomalies in lower-extremity joints, and any musculoskeletal diseases of the lower extremities.

### 2.4. Randomization

University female students (100) were assessed for eligibility; 47 were excluded from the trial because they did not meet the inclusion criteria, including 29 female students who had the toe-out angle, 18 with a BMI more than 24 kg/m^2^, and 3 subjects refused to participate. As a result, 50 female students were included in the current trial. They were randomly divided into two equal-sized groups. Four subjects did not complete the treatment protocol due to difficulty in transportation, and four did not attend the follow-ups.

**Figure 1:**
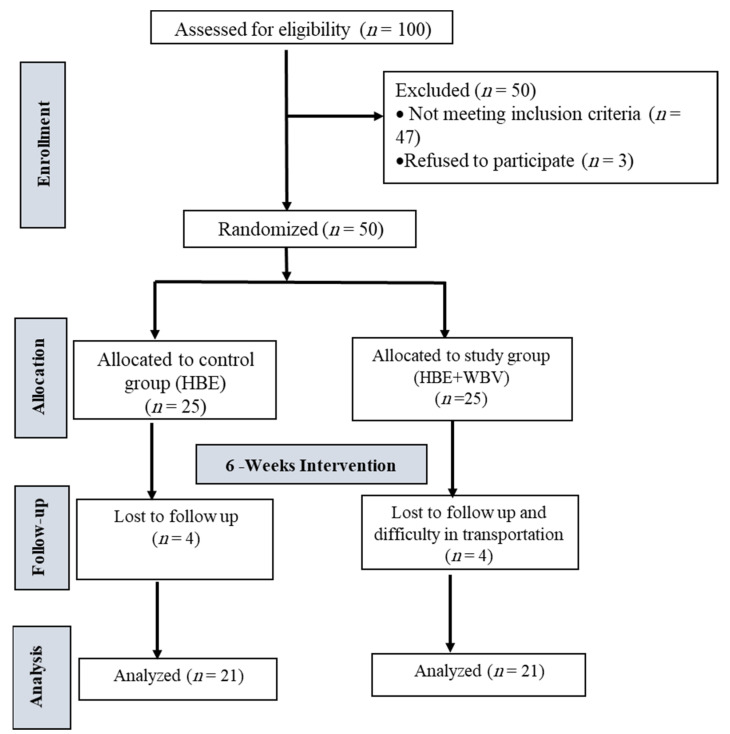
CONSORT flowchart.

The researcher applied computer-generated randomization to allocate subjects who met the inclusion criteria and consented to participate in the indicated groups. By using closed, dark envelopes, the assignment was masked. The same physical therapist carried out the two groups’ interventions. The randomized assignments were only known to the researcher and trainers. The assessors were not part of the exercise interventions and were kept blind about the randomization. The evaluation was carried out twice: once prior to the intervention (baseline) and once after six weeks of the intervention. The subjects in the control group (*n* = 21) performed only home-based exercise (HBE) after education, and those in the study group (*n* = 21) received WBV with HBE. Figure 1 represents randomization throughout the research.

## 3. Outcome Measures

### 3.1. Primary Outcome

**Figure 2:**
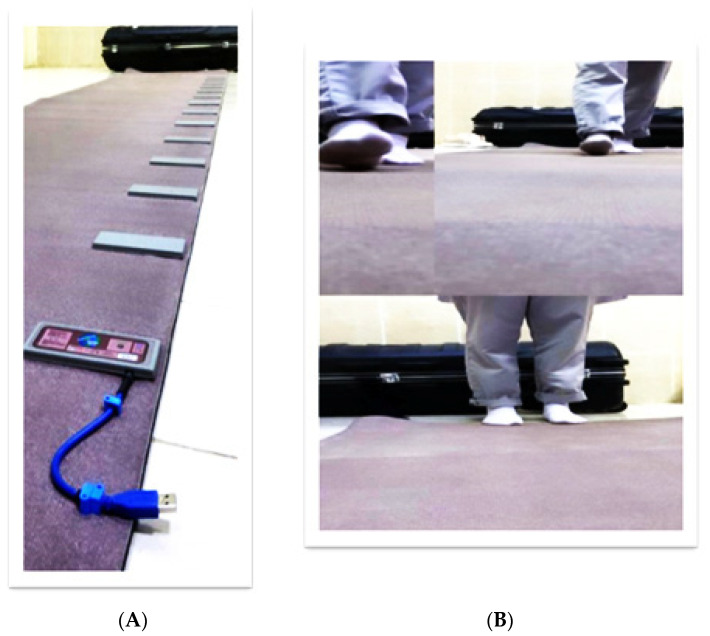
(**A**) Gait ride device. (**B**) Walking students using the gait ride device.

All participants have a toe-in degree based on data obtained from The GAIT Rite system. The GAIT Rite system consists of a transportable walkway equipped with pressure-activated sensors. The walkway records when the sensors are engaged and how far apart they are from one another. After that, it enters these data into application software to compute gait parameters, including the spatial and temporal for each footfall and the average of all the parameters. A pressure-sensitive walkway system (GAITRite-System, GS) and Moto gnosis Labs Software with a Microsoft Kinect Sensor (MKS) were both relied on to evaluate gait patterns; the device’s accuracy and dependability were certified by Webster et al. [25]. Each student was asked to walk normally as much as possible using the gait training device without target walking, as each student was asked to make three trials barefoot to ensure the normality of the walking pattern. According to spatiotemporal parameters extracted from this device, the positive sign indicated to toe out, and a negative sign indicated to toe in. The cases with a toe in degree (negative sign) were chosen according to the criteria of the selected cases, as shown in Figure 2.

### 3.2. Secondary Outcome

The Western Ontario and McMaster osteoarthritis index (WOMAC) was used to assess the functional level of participants to predict osteoarthritis (OA) [26]. It has 24 questions with a score range of 0 (best) to 4 (worst), and three subscales. There are five questions on the intensity of knee pain: during walking, ascending stairs, sleeping at night, resting, and standing (scored 0–20); two on stiffness: stiffness in the morning, evening (scored 0–8); and 17 on limitations of physical function: getting in and out of the car, going shopping, putting on socks, taking off socks, rising from bed, lying in bed, getting in and out of the bath, setting, getting on and off the toilet, doing light household duties (cooking, dusting), and doing heavy household duties (moving furniture) (scored 0–68). The WOMAC scores ranged from 0 to 96, with higher scores indicating severe OA. It has been proven by McConnell et al. [26] that there were many other favorable factors other than age that might contribute to an increase in the liability of younger athletes to pain and stiffness. Thus, the WOMAC index score could be a predictor index to the beginning of osteoarthritis among younger-aged female students. As reported by Cibulka et al. [27], OA results from “wear and tear”; athletes and young individuals use their joints more and consequently increase the level of risk. Moreover, Cameron et al. [28] revealed a significant difference in WOMAC scores in the group with foot deformities relative to those without.

## 4. Intervention

### 4.1. Whole-Body Vibration (WBV) Device

**Figure 3:**
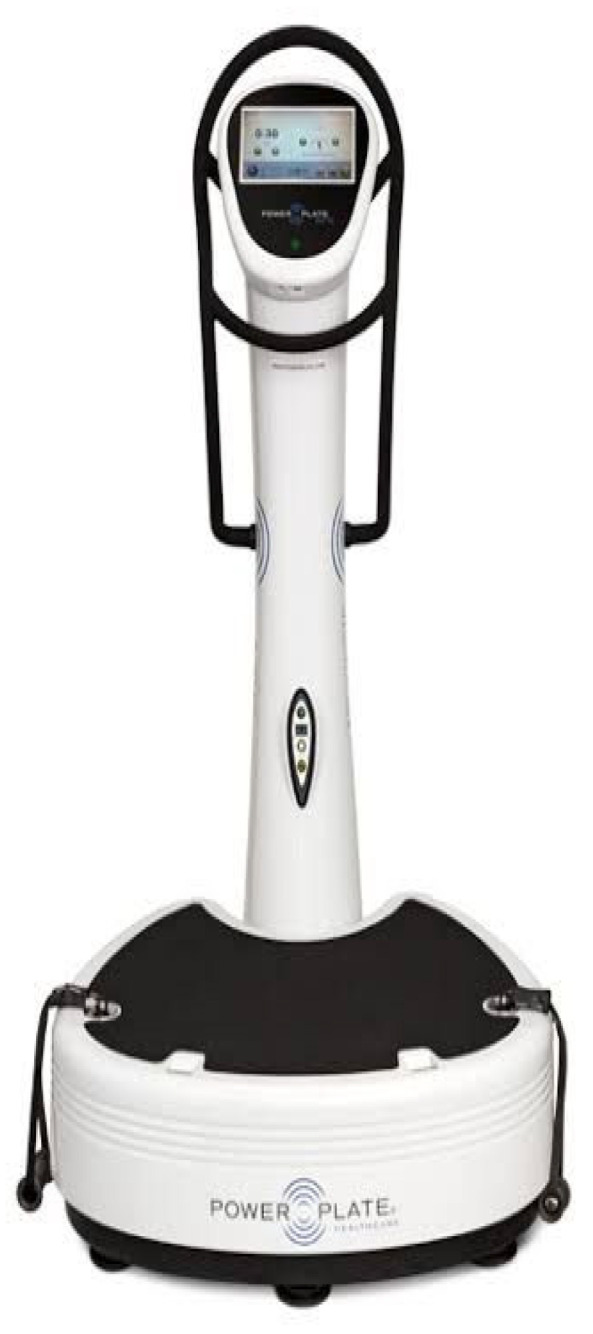
Whole-body vibration (WBV) device (Power Plate^®^ pro7HC™).

As shown in Figure 3, whole-body vibration (Power Plate^®^ pro7HC™) had low frequencies ranging from 5 to 15 Hz and an oscillating amplitude (2 mm). Five rehabilitation programs related to the ankle and foot for each case with toe-in (related to the degree of the toe angle) were selected. The exercise program’s purpose is to adjust the normal value of the toe angle degree to be varied within the normal degree. Excessive out-toeing can increase medial foot pressure, resulting in excessive subtalar joint pronation and lower-extremity problems. On the other hand, excessive lateral foot loading from in-toeing can over-supinate the foot, increasing the risk of a sprain to the lateral ligaments of the ankle and foot [27]. WBV rehabilitates and improves the neuromuscular performance of ankle and foot joints in healthy individuals [29]. These five sets were per session, three sessions per week for six weeks; according to the parameter device, 30 s for each case was documented. The five-rehabilitation program introduced by this device for ankle and foot was demonstrated as follows: (1) a hamstring stretch, where instead of hip rotation, stretch the leg on the platform, rotating the foot and hip in and out like a windshield wiper, as the chest is up and buttocks are back. (2) The squat matrix consists of a neutral stance, underhand grip, overhand grip, weights added while unsupported, three ranges of squat thresholds, including the initial range, mid-range, and deep/end range. (3) Dynamic hip movement, where the movement is driven through the hips and low back compression is avoided when reaching forward and driving hips as you lean back. (4) Feet are wide facing forward to maintain the correct posture and (5) the hip flexor stretch. In this procedure, the participants turn their back legs slightly inward and keep their heels down throughout the movement. Silva et al. [30] verified the whole-body vibration measurement device authentication.

### 4.2. Home-Based Exercise

Home-based exercise offers five different workout plans. Active range-of-motion, muscle-strengthening, and stretching movements made up the exercise protocol. The ankle and foot muscle strength, flexibility, and range of motion were to be increased as part of the procedure. Participants were guided to practice the exercise protocol [31]. The majority of the exercises recommended can be adopted into daily life. For instance, individuals were instructed to regularly dorsiflex and evert their ankles while sitting on a chair, and progressively straighten each leg out in front of them. Each exercise could be done up to ten times by each person, according to instructions. A thorough handout with instructions and pictures of the exercises was given to each subject. For six weeks, the subjects attended HBE sessions more than three times each week. The subjects were told that no exercise should cause them any pain.

## 5. Statistical Analysis

GraphPad Prism version 9 was used to analyze collected data, which were presented as the mean ± SEM. The difference in demographic characteristics among the HBE and HBE + WBV groups was detected using Student’s *t*-test. A two-way repeated-measures ANOVA was used to assess outcome variables between the two groups. The effect size between-group effect was calculated by a partial eta square. Significance was considered when *p* < 0.05. The paired *t*-test was performed to test the outcome difference within each group. Using a linear regression model with a generalized estimating equation (GEE) correction, the relationship between the degree of toe angle and the WOMAC index in both groups was evaluated at baseline and six weeks after the intervention. Any extremes were eliminated after the data were standardized. Before using the parametric assumption, the normality and homogeneity of the variance were statistically evaluated.

## 6. Results

Fifty female students were included in the current trial. All subjects met the inclusion criteria, being 19–21 years old, with a BMI of 20–24 kg/m^2^ and a height of 155–170 cm. All participants had a toe-in degree in at least one of the two feet based on data from The GAIT Rite system. The exclusion criteria were previous trauma, a history of surgery in both legs, congenital anomalies in the lower-extremity joints and any musculoskeletal diseases of the lower extremities.

The participants were randomly divided into two groups (the HBE group and the HBE + WBV group) (all *n* = 25, Figure 1). After the study process, four subjects in the HBE group lost follow-up, and four subjects in the HBE + WBV group did not complete the treatment protocol due to difficulty in transportation. The final sample analyzed included 42 participants (21 in the HBE group and 21 in the HBE + WBV group). Demographic data were obtained for the participants, as shown in Table 1. No significant difference in BMI (*p* = 0.33; η^2^ = 0.03) and age (*p* = 0.08; η^2^ = 0.07) was reported between the groups.

### 6.1. Toe Angle and WOMAC Index

**Figure 4:**
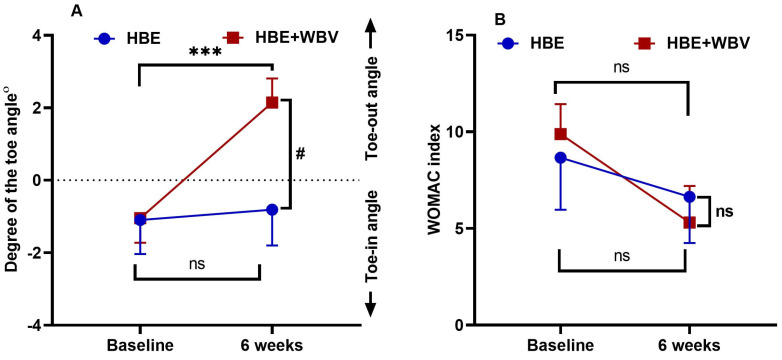
(**A**) Change in the degree of the toe angle over time in each group. Data are expressed as the mean ± SEM. *** *p* < 0.001 within the HBE + WBV group at baseline vs. after six weeks, # *p* < 0.05 vs. HBE group after six weeks. (**B**) Change in the WOMAC index over time in each group. HBE: home-based exercise; WBV: whole-body vibration exercise; WOMAC: Western Ontario and McMaster Universities Osteoarthritis index; ns: not significant.

Data for the WOMAC index and toe angle at baseline and six weeks after the intervention are shown in Table 2 and Figure 4. At baseline, participants in both groups (HBE and HBE + WBV) showed a toe-in angle. The degree of the angle displayed an insignificant difference after six weeks of intervention relative to the baseline in the HBE group (*p* = 0.11). Participants in the HBE + WBV group showed significant improvement in the toe-in angle after six weeks of intervention (*p* < 0.001) compared with baseline data. After the intervention, there was a significant improvement in the toe-in angle of HBE + WBV compared with that of the HBE group (*p* = 0.02).

HBE and HBE + WBV groups showed no significant decrease in the WOMAC index after six weeks of intervention compared with the baseline data (*p* = 0.25, 0.13). Additionally, compared to the HBE group, the HBE + WBV group’s WOMAC index revealed an insignificant decline. A two-way repeated-measures ANOVA showed significant change for the degree of the toe angle outcome variable, F (1, 40) = 4.09, *p* = 0.04, η^2^ = 0.13, but no significant change for the WOMAC index variable, F(1, 40) = 0.37; *p* = 0.12; η^2^ = 0.01.

### 6.2. Correlation between Toe Angle and WOMAC Index

**Figure 5:**
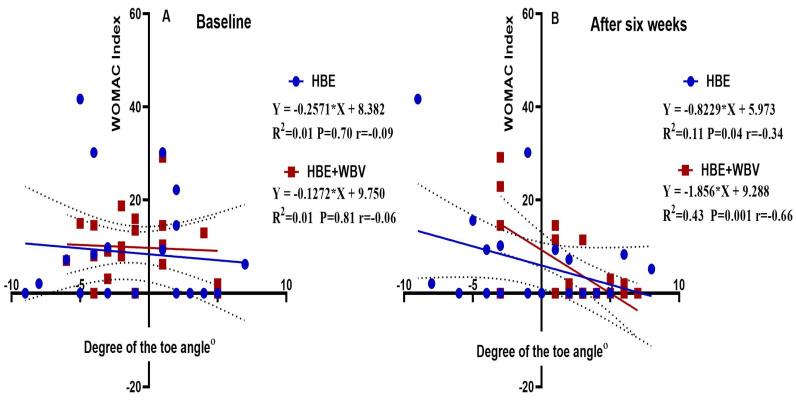
Linear regression of the degree of the toe angle versus the WOMAC index at baseline (**A**) and after six weeks of intervention (**B**).

Figure 5A demonstrates a weak negative correlation between the WOMAC index and the toe angle degree in both groups (HBE and HBE + WBV), (r = −0.09, −0.06) respectively, at baseline. The linear regression displayed statistical insignificance concerning this. In both groups (HBE and HBE + WBV), improvement of the toe angle explains 1% of the decrease in WOMAC index (*p* = 0.70, 0.81), respectively. Figure 5B demonstrates an intermediate negative correlation between WOMAC index and degree of the toe angle in both groups (HBE and HBE + WBV), (r = −0.34, −0.66) respectively, after six weeks of intervention. This was statistically significant in HBE + WBV, as indicated through linear regression analysis. In HBE, the angle improvement explains 11% of the decrease in the WOMAC index (*p* = 0.04). In HBE + WBV, the improvement of toe angle explains 43% of the decrease in the WOMAC index (*p* = 0.001).

## 7. Discussion

During walking, foot movements contribute to the movement of all lower-extremity bones. The foot serves as a base for motion, as well as joint realization and body positioning for balance. In addition to providing a base for support that enables adjustment to an uneven terrain, the foot’s motion, which includes dorsiflexion and plantarflexion, reduces energy consumption throughout the swing and stance phases of the gait cycle [32]. Using a paired sample *t*-test, the current study’s findings revealed a significant difference in the mean value of the toe angle degree of the WBV group. The degree of the toe angle was shifted in before using WBV, and then converted to be shifted out after using it. Moreover, there was a highly significant difference using an independent samples *t*-test between the control and study groups. The degree of the toe angle after using WBV was significantly improved regarding the toe degree of the control group.

The current results were consistent with those of Sierra-Guzmán et al. [33]. They evaluated the impact of a 6-week whole-body vibration training on an unstable surface on the electrical activity of the ankle muscles in recreational atheletes with chronic ankle instability. Their results revealed a dramatic improvement in reaction times for peroneus brevis, peroneus longus, and tibialis anterior for the vibration group compared with the control group. That means improving ankle eversion range of motion that influences the degree of the toe angle to be shifted in to out. The current study showed similar results in improving the degree of the toe-in and decreasing its value within the WBV group.

The present results were further supported by Kim et al. [34], which showed an increased ankle evertor strength in athletes who suffer from chronic ankle instability in response to a neuromuscular rehabilitation program. Twenty female hockey players participated in this study and were split into the control and case groups. According to their findings, the neuromuscular rehabilitation program quickly boosted eccentric evertor strength, which eventually helped improve the evertor strength of unstable ankles. The similarities between the neuromuscular rehabilitation program’s underlying mechanism and WBV were mentioned by Pollock et al. [10]. In addition, improving the evertor muscle strength had a linear effect on improving the degree of toe angle.

Our results were also consistent with Tan et al. [35], who looked at the impact of whole-body vibration on chronic ankle instability in terms of sensorimotor deficiencies in strength, joint position awareness, and muscle activation. Their findings showed that the activation of the gastrocnemius and tibialis anterior muscles increased. According to Jo et al. [13], the activity of the gastrocnemius and tibialis anterior muscles significantly increases, and the tibialis anterior muscle functions as an ankle joint fixator. In contrast, the gastrocnemius muscle is supportive during toe gripping that occurs during toe grip movement at the stance phase of walking. Furthermore, during the late stance phase of the gait cycle, when walking, the gastrocnemius muscle plays a crucial role in assisting with the propulsive force.

The physiological effects of whole-body vibration include improved neuromuscular function and tonic vibration reflexes. In people who have osteoarthritis, it reduces pain and modifies proprioceptive function [36]. These positive output results after using WBV directly have a good effect on correcting and controlling the normal function of the foot and toe during walking, according to the current study findings.

Guler et al. [37] revealed how pes planus and hallux valgus foot deformities affected the functional state in women with knee osteoarthritis. A hallux valgus angle greater than 21 degrees and a lateral talometatarsal angle greater than 4 degrees for the pes planus were considered. In order to determine the functional foot disturbance objectively, the Western Ontario and McMaster Universities Osteoarthritis Index (WOMAC) was utilized to investigate two groups: one with foot deformity in one or both feet, and another with no deformity observed. A significant difference in the WOMAC scores in the group with foot deformities relative to those without was observed. Additionally, enhanced levels of disability in women with knee osteoarthritis and foot deformities were reported. The current study produced comparable findings to those reported earlier, with the WOMAC index declining as the toe angle increased, as seen in Figure 5.

Corum et al. [38] explored how whole-body vibration training affected the quality of life and isokinetic muscle performance in female patients with patellofemoral discomfort. Forty women participated in the study and were divided into two groups: the WBV group, which received WBV training in addition to home exercise; and the control group, which just exercised at home. The findings revealed that home exercise and eight weeks of WBV training were more effective than a control group in lowering pain and boosting knee extensor endurance.

Limitations of the current research were potential selection bias due to the study’s design. There were also potential influencing factors on the degree of the toe angle, such as physical activity levels or prior injuries. Functional variation among the participants can be added as a contributing limited factor.

## 8. Conclusions

It can be concluded that using the whole-body vibration training program was useful and positively affected the degree of the toe angle. Adjusting the toe angle affected the knee joint’s mechanics during walking. Thus, designing a (WBV) rehabilitation program was a proactive step in the physical therapy field for female adults with toe-in walking to improve their gait pattern and reduce subsequent neighbouring joint injuries such as osteoarthritis. According to the findings of the current results, we can predict the musculoskeletal foot and ankle disorders among the younger female age group, enabling orthopedists to introduce an early controlled rehabilitation program for their foot’s small intrinsic muscle group, thus improving the toe angle degree.

## Figures and Tables

**Table 1:** Demographic characteristics of the two groups’ participants.

Characteristic	HBE Group (*n* = 21)	HBE + WBV Group (*n* = 21)	*p*-Value	Effect Size (η^2^)
Age (yr)	20.76 ± 0.32	21.48 ± 0.22	0.08	0.07
BMI (kg/m^2^)	22.35 ± 0.89	23.50 ± 0.74	0.33	0.03

Values are shown as the mean ± standard error of the mean (SEM); BMI: body mass index; *p*-values are from Student’s *t*-test; *p* < 0.05 is statistically significant; η^2^: partial eta square.

**Table 2:** Outcome data for the degree of the toe angle and WOMAC index at baseline and after six weeks of intervention.

Variable	HBE Group				HBE + WBV Group				Group X Time Interaction *p*-Value	*P^b^*^)^ between Groups	Effect Size (η^2^)
	Baseline	After Six Weeks	MD (95%CI)	*P^a^*^)^ within Group	Baseline	After Six Weeks	MD (95%CI)	*P^a^*^)^ within Group			
Toe angle	−1.10 ± 0.94	−0.81 ± 0.99	0.29 (−0.07–0.64)	0.11	−1.05 ± 0.68	2.14 ± 0.67	3.19 (1.59–4.79)	<0.001	0.04	0.02	0.13
WOMAC index	8.66 ± 2.70	6.64 ± 2.39	−2.03 (−5.61–4.56)	0.25	9.89 ± 1.56	5.31 ± 1.89	−4.57 (−10.68–1.53)	0.13	0.12	0.66	0.01

Values are shown as the mean ± standard error of the mean (SEM); HBE: home-based exercise; WBVE: whole-body vibration exercise; WOMAC: Western Ontario and McMaster Universities Osteoarthritis index; MD: mean difference; CI: confidence interval; *P^a^*^)^-value within the group from the paired samples *t*-test; *P^b^*^)^-value between the HBE and HBE + WBV groups after six weeks from independent sample *t*-test; η^2^: partial eta square between the HBE and HBE + WBV groups after six weeks; Group X time interaction *p*-value from a two-way repeated-measures ANOVA.

## Data Availability

The datasets used and/or analyzed during the current study are available from the corresponding author upon reasonable request.

## List of Abbreviations

WBV	Whole-body vibration
HBE	Home-based exercise
WOMAC index	Western Ontario and McMaster osteoarthritis index
CONSORT	Consolidated Standards of Reporting Trials
MKS	Microsoft Kinect Sensor
OA	Osteoarthritis
GEE	Generalized estimating equations
η2	eta squared

## References

[1] Chung K., Chung H., Halliday N. (2012). Gross Anatomy.

[2] Abd-Eltawab A.E., Ameer M.A., Eladl M.A., El-Sherbiny M., Ebrahim H.A., Elsherbini D.M.A. (2022). Sexual dimorphism impact on the ground reaction force acting on the mediolateral direction during Level walking: Hip abductor muscle biomechanics and its correlation to GRF moment arm. Front. Bioeng. Biotechnol..

[3] Al-kharaz A.A., Chong A.K. (2022). Gender differences in ankle kinematics of adults during gait. J. Sex-Gend.-Specif. Med..

[4] Ristolainen L., Heinonen A., Waller B., Kujala U.M., Kettunen J.A. (2009). Gender differences in sport injury risk and types of inju-ries: A retrospective twelve-month study on cross-country skiers, swimmers, long-distance runners and soccer players. J. Sports Sci. Med..

[5] Quatman C.E., Ford K.R., Myer G.D., Paterno M.V., Hewett T.E. (2008). The effects of gender and pubertal status on generalized joint laxity in young athletes. J. Sci. Med. Sport..

[6] Fukano M., Fukubayashi T., Banks S.A. (2018). Sex differences in three-dimensional talocrural and subtalar joint kinematics during stance phase in healthy young adults. Hum. Mov. Sci..

[7] de Sire A., Lippi L., Ammendolia A., Cisari C., Venetis K., Sajjadi E., Fusco N., Invernizzi M. (2021). Physical exercise with or without whole-body vibration in breast cancer patients suffering from aromatase inhibitor—Induced musculoskeletal symptoms: A pilot randomized clinical study. J. Pers. Med..

[8] Cloak R., Nevill A., Day S., Wyon M. (2013). Six-week combined vibration and wobble board training on balance and stability in footballers with functional ankle instability. Clin. J. Sport Med..

[9] Cardinale M., Bosco C. (2003). The use of vibration as an exercise intervention. Exerc. Sport Sci. Rev..

[10] Pollock R.D., Woledge R.C., Martin F.C., Newham D.J. (2012). Effects of whole body vibration on motor unit recruitment and threshold. J. Appl. Physiol..

[11] Rendos N.K., Jun H.-P., Pickett N.M., Lew Feirman K., Harriell K., Lee S.Y., Signorile J.F. (2017). Acute effects of whole body vibration on balance in persons with and without chronic ankle instability. Res. Sports Med..

[12] Chang W.-D., Chen S., Tsou Y.-A. (2021). Effects of whole-body vibration and balance training on female athletes with chronic ankle instability. J. Clin. Med..

[13] Jo Y.-R., Jeong M.-B., Lee D.-W. (2018). The effect of whole body vibration exercise on ankle joint spasticity patients with chronic stroke. J. Korean Phys. Ther..

[14] Hosea T.M., Carey C.C., Harrer M.F. (2000). The gender issue: Epidemiology of ankle injuries in athletes who participate in basketball. Clin. Orthop. Relat. Res..

[15] Brumitt J., Mattocks A., Loew J., Lentz P. (2020). Preseason functional performance test measures are associated with injury in female college volleyball players. J. Sport Rehab..

[16] McAlindon T.E., LaValley M.P., Harvey W.F., Price L.L., Driban J.B., Zhang M., Ward R.J. (2017). Effect of intra-articular triamcinolone vs saline on knee cartilage volume and pain in patients with knee osteoarthritis: A randomized clinical trial. JAMA.

[17] Sandler E.B., Condon K., Field-Fote E.C. (2021). Efficacy of transcutaneous spinal stimulation versus whole body vibration for spasticity reduction in persons with spinal cord injury. J. Clin. Med..

[18] Qiu C.G., Chui C.S., Chow S.K.H., Cheung W.-H., Wong R.M.Y. (2022). Effects of whole-body vibration therapy on knee Osteoarthritis: A systematic review and meta-analysis of randomized controlled trials. J. Rehabil. Med..

[19] Gonçalves de Oliveira R., Coutinho H.M.E.L., Martins M.N.M., Bernardo-Filho M., de Sá-Caputo D.d.C., Campos de Oliveira L., Taiar R. (2023). Impacts of whole-body vibration on muscle strength, power, and endurance in older adults: A systematic review and meta-analysis. J. Clin. Med..

[20] Amoako A.O., Pujalte G.G.A. (2014). Osteoarthritis in young, active, and athletic individuals. Clin. Med. Insights Arthritis Musculoskelet. Disord..

[21] Moher D., Hopewell S., Schulz K.F., Montori V., Gøtzsche P.C., Devereaux P.J., Elbourne D., Egger M., Altman D.G. (2012). CONSORT 2010 explanation and elaboration: Updated guidelines for reporting parallel group randomised trials. Int. J. Surg..

[22] Wang P., Yang L., Liu C., Wei X., Yang X., Zhou Y., Jiang H., Lei Z., Reinhardt J.D., He C. (2016). Effects of whole body vibration exercise associated with quadriceps resistance exercise on functioning and quality of life in patients with knee osteoarthritis: A randomized controlled trial. Clin. Rehabil..

[23] Park Y.G., Kwon B.S., Park J.-W., Cha D.Y., Nam K.Y., Sim K.B., Chang J., Lee H.J. (2013). Therapeutic effect of whole body vibration on chronic knee osteoarthritis. Ann. Rehabil. Med..

[24] Lai Z., Lee S., Hu X., Wang L. (2019). Effect of adding whole-body vibration training to squat training on physical function and muscle strength in individuals with knee osteoarthritis. J. Musculoskelet. Neuron Interact..

[25] Webster K.E., Wittwer Je Fau-Feller J.A., Feller J.A. (2005). Validity of the GAITRite walkway system for the measurement of averaged and individual step parameters of gait. Gait Posture.

[26] McConnell S., Kolopack P., Davis A.M. (2001). The Western Ontario and McMaster Universities Osteoarthritis Index (WOMAC): A review of its utility and measurement properties. Arthritis Rheum..

[27] Cibulka M.T., Winters K., Kampwerth T., McAfee B., Payne L., Roeckenhaus T., Ross S.A. (2016). Predicting foot progression angle during gait using two clinical measures in healthy adults, a preliminary study. Int. J. Sports Phys. Ther..

[28] Cameron K.L., Hsiao M.S., Owens B.D., Burks R., Svoboda S.J. (2011). Incidence of physician-diagnosed osteoarthritis among active duty United States military service members. Arthritis Rheum..

[29] Rehn B., Lidström J., Skoglund J., Lindström B. (2007). Effects on leg muscular performance from whole-body vibration exercise: A systematic review. Scand. J. Med. Sci. Sports.

[30] Silva P., Seabra E., Mendes J. (2022). Design, development, and validation of a whole-body vibration measurement device. ASME Open J. Eng..

[31] Fitzgerald G.K., Oatis C. (2004). Role of physical therapy in management of knee osteoarthritis. Curr. Opin. Rheumatol..

[32] Chan C.W., Rudins A. (1994). Foot Biomechanics during Walking and Running. Mayo Clinic Proceedings.

[33] Sierra-Guzmán R., Jiménez J.F., Ramírez C., Esteban P., Abián-Vicén J. (2017). Effects of synchronous whole body vibration training on a soft, unstable surface in athletes with chronic ankle instability. Int. J. Sports Med..

[34] Kim T., Kim E., Choi H. (2017). Effects of a 6-week neuromuscular rehabilitation program on Ankle-Evertor strength and postural stability in elite women field hockey players with chronic ankle instability. J. Sport Rehab..

[35] Tan J., Wu X., Clark C.C., Barton V., Chen S., Liu S., Zhou X., Xu C., Ma T., Qi B. (2022). The effect of whole body vibration on sensorimotor deficits in people with chronic ankle instability: A systematic review and meta-analysis. Clin. Rehabil..

[36] Bonanni R., Cariati I., Romagnoli C., D’Arcangelo G., Annino G., Tancredi V. (2022). Whole body vibration: A valid alternative strategy to exercise?. J. Funct. Morphol. Kinesiol..

[37] Guler H., Karazincir S., Turhanoglu A.D., Sahin G., Balci A., Ozer C. (2009). Effect of coexisting foot deformity on disability in women with knee osteoarthritis. J. Am. Podiatr. Med. Assoc..

[38] Corum M., Basoglu C., Yakal S., Sahinkaya T., Aksoy C. (2018). Effects of whole body vibration training on isokinetic muscular performance, pain, function, and quality of life in female patients with patellofemoral pain: A randomized controlled trial. J. Musculoskelet. Neuron Interact..

